# Supplementary material for: A Comprehensive Mass Spectrometry-Based Workflow for Clinical Metabolomics Cohort Studies
Source: Metabolites. 2022 Nov 24;12(12):1168. doi: 10.3390/metabo12121168 (PMC9782571; doi:10.3390/metabo12121168)
Supplement: Supplementary file 1 [file metabolites-12-01168-s001.zip › metabolites-1953099-supplementary.pdf]

A Comprehensive Mass Spectrometry-Based Workflow for Clinical Metabolomics Cohort Studies

Zhan Shi [1,†], Haohui Li [1,†], Wei Zhang [2], Youxiang Chen [2], Chunyan Zeng [2], Xiuhua Kang [2], Xinping Xu [2],
Zhenkun Xia [3], Bei Qing [3], Yunchang Yuan [3], Guodong Song [4], Camila Caldana [5], Junyuan Hu [1,*],
Lothar Willmitzer [5,*] and Yan Li [1,*]

[1] Metanotitia Inc., No 59. Gaoxin South 9th Road, Yuehai Street, Nanshan District, Shenzhen 518056, China; zhan.shi@metanotitia.com (Z.S.); haohui.li@metanotitia.com (H.L.)
[2] The First Affiliated Hospital of Nanchang University, No.17 Yongwaizheng Street, Nanchang 330209, China
[3] The Second Xiangya Hospital of Central South University, Furong District, Changsha 410011, China;
[4] The Second Hospital of Tianjin Medical University, No 23. Pingjiang Road, Hexi District, Tianjin 300211, China
[5] Max Planck Institute of Molecular Plant Physiology, Potsdam Science Park, Am Muehlenberg 1, 14476 Potsdam, Germany
* Correspondence: junyuan.hu@metanotitia.com (J.H.); willmitzer@mpimp-golm.mpg.de (L.W.); yan.li@metanotitia.com (Y.L.)
† These authors contributed equally to this work.

File S1. Standard operation protocol for biospecimen collection and storage.

Prior to sample collection, all health care professionals were trained on sites and operated by the same standard operation protocol (SOP) listed below.

I. Informed consent

a. The patient must sign two copies of the written informed consent, one for the patient and one for the clinic as a record.
b. Clinical research associate (CRA) should assign the patient a unique ID and mark the informed consent with the prepared bar-code label, then record basic information of the patient, i.e., name, sex, age.

II. Pre-collection procedure (one day before blood collection)

a. CRA should print the bar-code labels for the tubes according to the labels on the informed consents, pre-cool the centrifuge machine, and prepare the equipment (4°C pre-cooled blood transportation box, blood collection needle, one 6-mL ethylenediaminetetraacetic acid (EDTA) coated tube for each patient).

   CRA should inform patients to fast and stop drinking water from the night before blood harvest, and to avoid any critical physical activity, spicy or greasy food, alcohol, and caffeine.

III. Blood samples

1. Blood collection
   a. Timing: fasted blood should be collected in the morning before breakfast and exercises.
   b. Vein selection: let the patient be seated for 3-4 min, then place the forearm horizontally on the cushion, choose the elbow vein or the vein on the back of the hand that is easy to fix and visible. Should not take blood immediately after prolonged vein occlusion.
   c. Volume: 5 mL of blood for each 6-mL EDTA tube for each patient. The EDTA-tube should be inverted several times immediately after collection.
   d. Recording: CRA should fill out the "Blood sample collection record" form.
   e. Transportation: blood samples should be transported using the 4°C pre-cooled blood transportation box to the clinic lab.

f. Record uploading: each day the CRA should upload the "Blood sample collection record" form into the archive.

2. Plasma separation

a. Centrifuge: blood sample should be centrifuged at 3,800 rpm for 12 min at 4ºC. The blood samples in the EDTA-tube should be separated within 30 min, if not, temporally store at 4ºC.

b. Aliquoting: After centrifugation, transfer the blood collection tube to the test tube rack. During the transfer process, handle it gently to avoid disturbing the white blood cell layer. The upper clear liquid is plasma. Use a pipette to disperse the upper clear plasma into two 2-mL sterile cryopreservation tubes and two 2-mL EP tubes that are pre-cooled and labeled with barcodes in advance. Aliquots about 1 mL for each cryopreservation tube, and 100 μL for each EP tube. Try not to touch the cell layer. Discard remains.

c. Storage: store at liquid nitrogen tank or at -80ºC freezer. Avoid repeated freezing and thawing.

d. Storage identification and recording: Plasma samples need to record the storage location, including cryopreservation boxes, freezers, refrigerators, and liquid nitrogen tanks to ensure the uniqueness and traceability of the sample storage location to facilitate future sample search. Record the storage location of the plasma sample in the "Sample Storage Record Form".

File S2. Composition of QC mixes (QC_{mix} for LC-MS polar was set as Broadhurst et al guided).

FAME mix for GC-MS [1]	QC_{mix} for GC-MS [2]	QC_{mix} for LC-MS polar [3]	QC_{mix} for LC-MS lipid
Methyl octanoate	4-Hydroxyproline	Abscisic acid	LIPIDOMIX ®
Methyl nonanoate	Alanine	Azelaic acid	
Methyl decanoate	alpha-Ketoglutarate	(+)-Catechin	
Methyl dodecanoate	alpha-Tocopherol	Dexamethasone	
Methyl tetradecanoate	Arachidic acid	Gibberellic acid A3	
Methyl palmitate	Asparagine	Glycyl-L-phenylalanine	
Methyl stearate	Aspartic acid	L-Histidinol	
Methyl arachidate	Chlorogenic acid	L-Kynurenine	
Methyl behenate	Cholesterol	Nicotinuric acid	
Methyl tetracosanoate	Valine	Phenaceturic acid	
Methyl hexacosanoate	Sucrose		
Methyl octacosanoate	D-(+)-Glucose		
Methyl triacontanoate	Glucose-6-phosphate		
	Glutamic acid		
	Shikimic acid		
	Lysine		
	Methionine		
	N-Acetylaspartic acid		
	Nicotinic acid		
	Putrescine		
	Pyruvate		

	Salicylic acid		
	Serine		

File S3. LC-MS acquisition parameters.

Analysis	Parameters	LC-MS polar	LC-MS lipid
Chromatography	Column	ACQUITY HSS T3 column (2.1 × 100 mm, 1.8 µm, Waters, Milford, MAUK) coupled to a an ACQUITY HSS T3 VanGuard precolumn (2.1 × 5 mm, 1.8 µm)	ACQUITY BEH C8 column (2.1 ×100 mm, 1.7 µm, Waters, Milford, MA) coupled to an ACQUITY BEH C8 VanGuard precolumn (2.1 × 5 mm, 1.7 µm, Waters, Milford, MA)
	Colum temperature, °C	40	60
	Autosampler temperature, °C	10	10
	Mobile phase buffer A	Water containing 0.1% formic acid	Water containing 0.1% acetate acid and 10 mM ammonium acetate
	Mobile phase buffer B	100% Acetonitrile containing 0.1% formic acid	Acetonitrile/isopropanol (v/v, 7:3) solution containing 0.1% acetate acid and 10 mM ammonium acetate
	Mobile phase gradient separation	0-1 min 1% B, 1-11 min linear gradient from 1 to 40% B, 11-13 min linear gradient from 40-70% B, 13-15 min linear gradient from 70-99% B, 15-18 min 99% B, 18-19 min 99% B, 19-22 min 1% B, re-equilibrate with 1% buffer B	0-1 min 55% B, 1-4 min linear gradient from 45 to 75% B, 4-12 min linear gradient from 75-89% B, 12-15 min linear gradient from 89-100% B, 15-19.5 min 100% B, and 19.51-24 min 55% B, re-equilibrate with 55% buffer B
	Mobile phase flow rate, µL/min	400	400
Mass spectrometry (Thermo-Fisher Q-Exactive (Bremen, Germany))	Mass range, m/z	100-1500	100-1500
	Sheath gas flow rate, Arb	30	30
	Aux gas flow rate, Arb	10	10

	Sweep gas flow rate, Arb	0	0
	Spray voltage, kV	3.8	4
	Capillary temperature	350	350
	S-lens RF level	50	50
	Aux gas heater temperature	320	350
Full MS	Resolution	70,000 (FWHM, 200 m/z)	
	AGC target	3.00E+06	
	Maximum IT, ms	200	
	Spectrum data type	Profile	
Data dependent MS/MS	Resolution	17,500 (FWHM, 200 m/z)	
	AGC target	1.00E+05	
	Maximum IT, ms	50	
	Loop count	5	
	TopN	5	
	Isolation window, m/z	1.5	
	Fixed first mass	Dynamic first mass	
	(N)CE/stepped (N)CE	30	
	Spectrum data type	Profile	

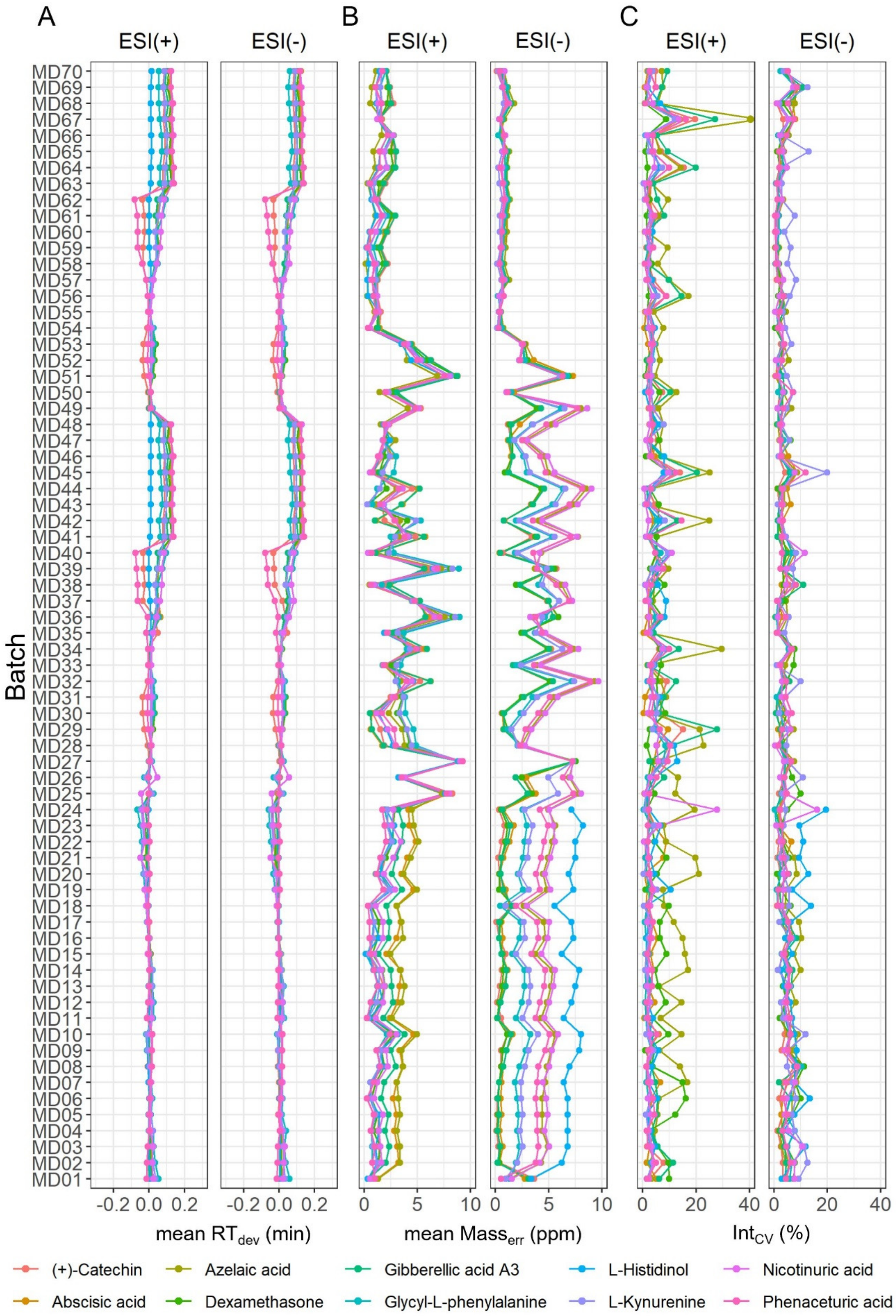

A
B
C
ESI(+)
ESI(-)
Batch
mean RT_dev (min)
mean Mass_err (ppm)
Int_CV (%)
(+)-Catechin
Abscisic acid
Azelaic acid
Dexamethasone
Gibberellic acid A3
Glycyl-L-phenylalanine
L-Histidinol
L-Kynurenine
Nicotinuric acid
Phenaceturic acid

Figure S1. LC-MS polar chromatographic stability and Orbitrap mass accuracy monitored by QC mixes.

A, mean RT_{dev} (retention time deviation). B, mean $Mass_{err}$ (mass error). C, Int_{CV} (intensity coefficient of variation). Data were acquired from 70 batches in positive (ESI (+)) and negative (ESI (-)) ionization modes separately.

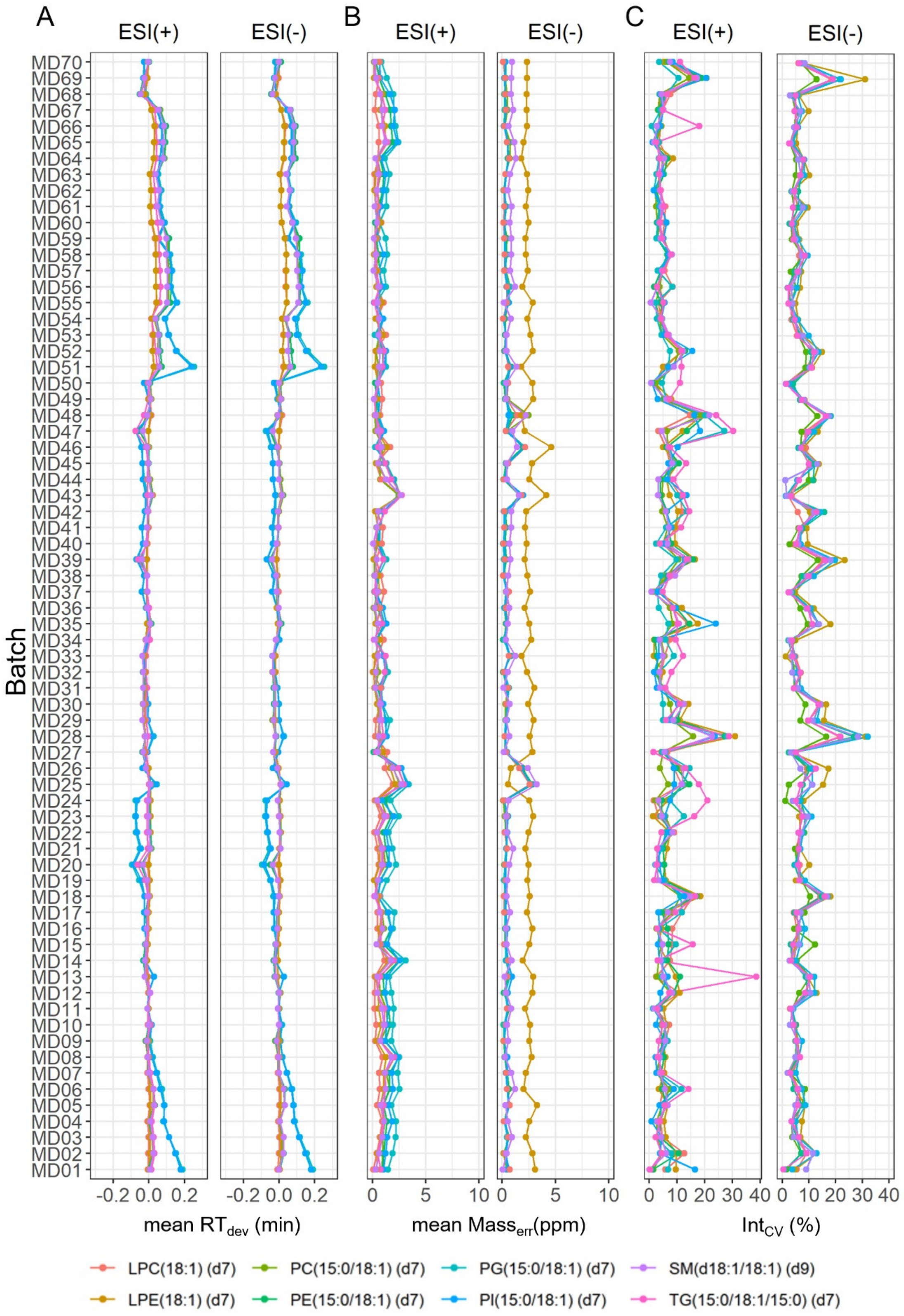

A
B
C
ESI(+)
ESI(-)
Batch
MD70
MD01
mean RT$_{dev}$ (min)
mean Mass$_{err}$(ppm)
Int$_{CV}$ (%)
LPC(18:1) (d7)
PC(15:0/18:1) (d7)
PG(15:0/18:1) (d7)
SM(d18:1/18:1) (d9)
LPE(18:1) (d7)
PE(15:0/18:1) (d7)
PI(15:0/18:1) (d7)
TG(15:0/18:1/15:0) (d7)

Figure S2. LC-MS lipid chromatographic stability and Orbitrap mass accuracy monitored by QC mixes.

A, mean RT_{dev} (retention time deviation). B, mean $Mass_{err}$ (mass error). C, Int_{CV} (intensity coefficient of variation). Data were acquired from 70 batches in positive (ESI (+)) and negative (ESI (-)) ionization modes separately.

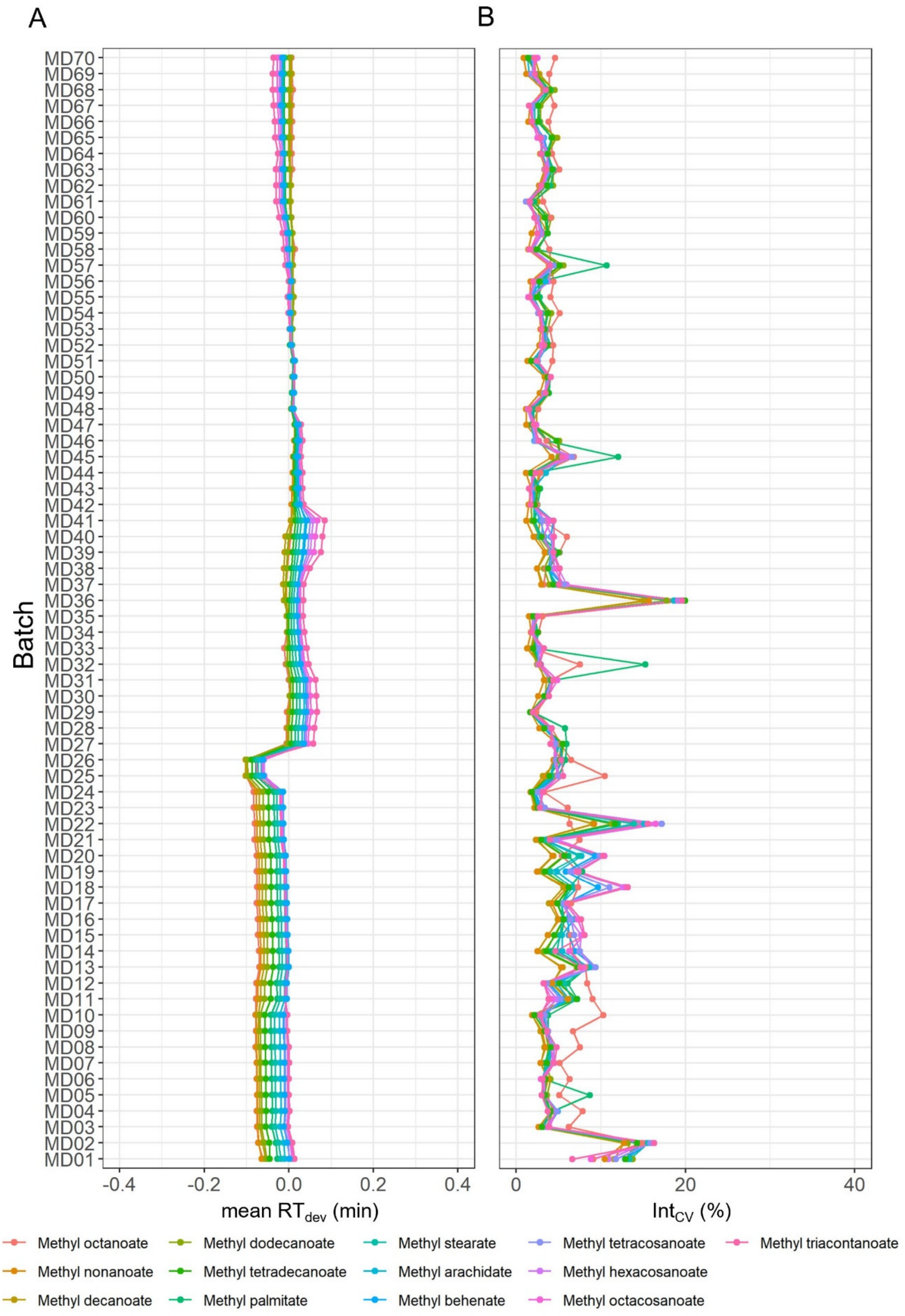
A
B
Batch
MD70
MD69
MD68
MD67
MD66
MD65
MD64
MD63
MD62
MD61
MD60
MD59
MD58
MD57
MD56
MD55
MD54
MD53
MD52
MD51
MD50
MD49
MD48
MD47
MD46
MD45
MD44
MD43
MD42
MD41
MD40
MD39
MD38
MD37
MD36
MD35
MD34
MD33
MD32
MD31
MD30
MD29
MD28
MD27
MD26
MD25
MD24
MD23
MD22
MD21
MD20
MD19
MD18
MD17
MD16
MD15
MD14
MD13
MD12
MD11
MD10
MD09
MD08
MD07
MD06
MD05
MD04
MD03
MD02
MD01
-0.4
-0.2
0.0
0.2
0.4
mean RT_{dev} (min)
0
20
40
Int_{CV} (%)
Methyl octanoate
Methyl nonanoate
Methyl decanoate
Methyl dodecanoate
Methyl tetradecanoate
Methyl palmitate
Methyl stearate
Methyl arachidate
Methyl behenate
Methyl tetracosanoate
Methyl hexacosanoate
Methyl octacosanoate
Methyl triacontanoate

Figure S3. GC-MS chromatographic stability monitored by FAME.

A, mean RT_{dev} (retention time deviation). B, Int_{CV} (intensity coefficient of variation). Data were acquired from 70 batches across studies.

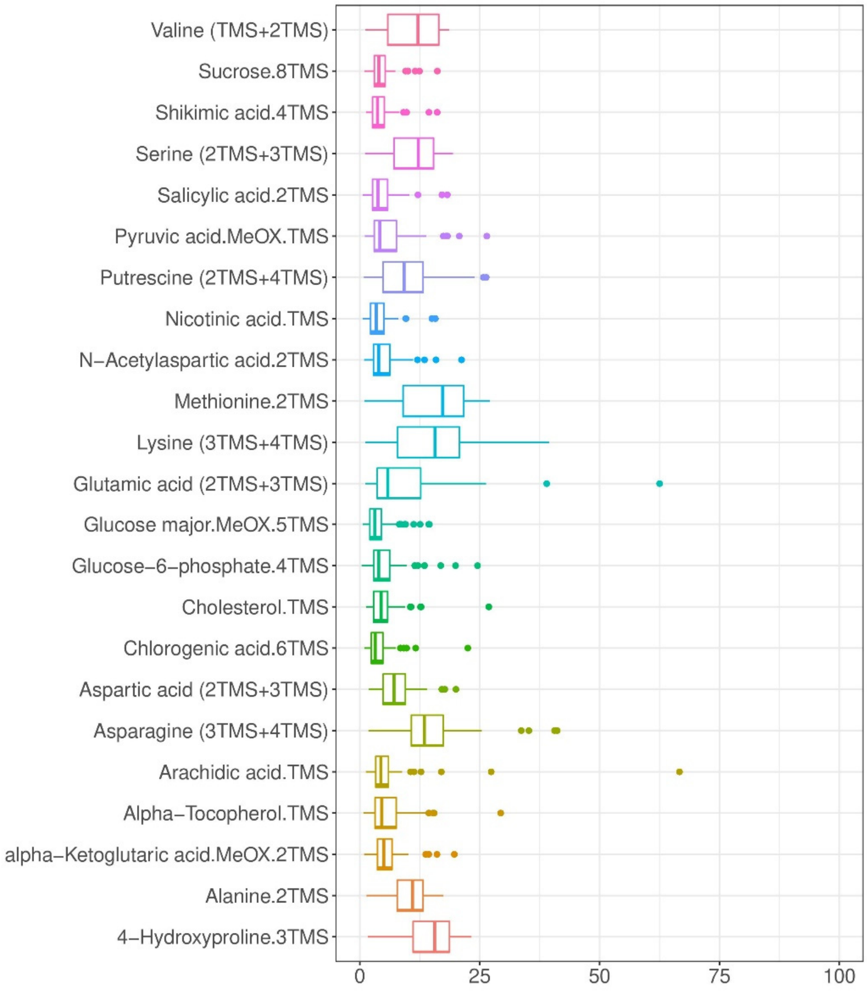

Figure S4. GC-MS chromatographic stability monitored by QC mixes.

Box plots represent the intensity coefficient of variation (CV) of each reference compound. Data were acquired from 70 batches.

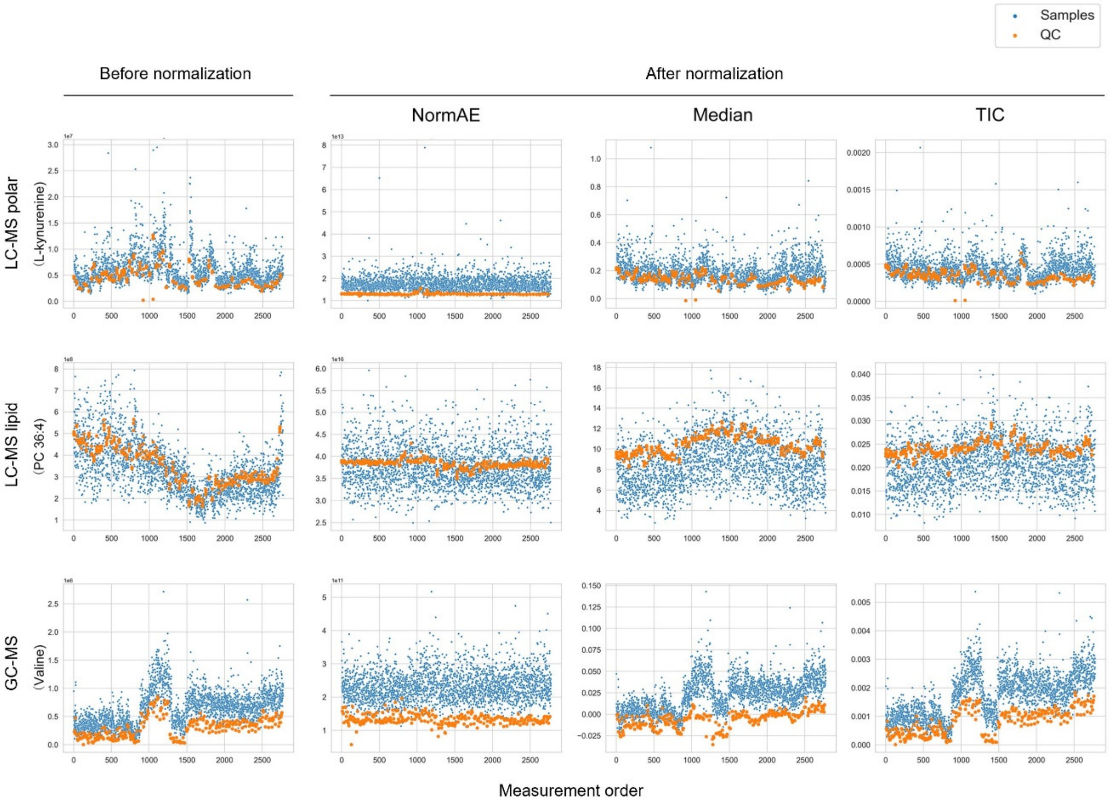

Figure S5. Normalization performance.

Intensities of representative features (LC-MS polar: L-kynurenine; LC-MS lipid: PC 36:4; GC-MS: valine) before and after normalization with NormAE, median and TIC. Orange dots: QC_{NIST}; Blue dots: biological samples. For each graph, the x-axis represents the measurement order, and the y-axis represents the feature intensity.

Table S1. The demographics of the CRC sub-cohort study.

TJH: The Second Hospital of Tianjin Medical University; NCH: First Affiliated Hospital of Nanchang University (Nanchang, Jiangxi Province); XYH: Second Xiangya Hospital of Central South University (Changsha, Hunan Province); HCtrl: Healthy Control; CRC, colorectal cancer.

**Demographics**		**HCtrl**	**CRC**
n		236	279
Hospital, n (%)	TJH	-	17 (5.9)
	NCH	128 (54.2)	259 (92.8)
	XYH	108 (45.8)	3 (1.1)
Mean age (SD)		45.6 (13.4)	60.2 (12.0)
Female (%)		124 (52.5)	109 (39.1)
Diabetes (%)		-	22 (8.0)
Hypertension (%)		-	74 (26.8)
Smoking History (%)	Never	171 (72.5)	202 (73.7)
	Current	57 (24.2)	53 (19.3)
	Used to	8 (3.4)	19 (6.9)
Alcohol-drinking History (%)	Never	153 (64.8)	220 (80.6)
	Current	83 (35.2)	50 (18.3)
	Used to	-	3 (1.1)

References

[1] Kind, T.; Wohlgemuth, G.; Lee, D.Y.; Lu, Y.; Palazoglu, M.; Shahbaz, S.; Fiehn, O. FiehnLib: Mass Spectral and Retention Index Libraries for Metabolomics Based on Quadrupole and Time-of-Flight Gas Chromatography/Mass Spectrometry. *Anal. Chem.* **2009**, *81*, 10038–10048. https://doi.org/10.1021/ac9019522.

[2] Fiehn, O. Metabolomics by gas chromatography-mass spectrometry: Combined targeted and untargeted profiling. *Curr. Protoc. Mol. Biol.* **2016**, *114*, 30.4.1–30.4.32. https://doi.org/10.1002/0471142727.mb3004s114.

[3] Broadhurst, D.; Goodacre, R.; Reinke, S.N.; Kuligowski, J.; Wilson, I.D.; Lewis, M.R.; Dunn, W.B. Guidelines and considerations for the use of system suitability and quality control samples in mass spectrometry assays applied in untargeted clinical metabolomic studies. *Metabolomics* **2018**, *14*, 72. https://doi.org/10.1007/s11306-018-1367-3.
